# Understanding the Value of International Research Networks: An Evaluation of the International Cancer Screening Network of the US National Cancer Institute

**DOI:** 10.1200/JGO.19.00197

**Published:** 2019-10-10

**Authors:** Amanda L. Vogel, Douglas M. Puricelli Perin, Ya-Ling Lu, Stephen H. Taplin

**Affiliations:** ^1^ Frederick National Laboratory for Cancer Research Sponsored by the National Cancer Institute, Frederick, MD; ^2^ National Institutes of Health Library, National Institutes of Health, Bethesda, MD; ^3^ National Cancer Institute, National Institutes of Health, Bethesda, MD

## Abstract

**PURPOSE:**

International research networks have the potential to accelerate scientific progress via knowledge sharing and collaboration. In 2018, the US National Cancer Institute evaluated the International Cancer Screening Network (ICSN), in operation since 1988.

**METHODS:**

ICSN hosts a biennial scientific meeting and scientific working groups. A survey was fielded to 665 ICSN participants, and a bibliometric analysis was conducted for ICSN publications.

**RESULTS:**

A total of 243 individuals completed the survey (36.5%). They reported that participating in the ICSN helped advance their knowledge of cancer screening research (75.7%), policy development (56%), and implementation (47.7%). Approximately three-quarters agreed that ICSN facilitated knowledge sharing and networking among researchers and implementers (79.9%) and those working on different continents (74.0%) and cancer sites (73.7%). More than half reported that participating helped them form new collaborations in screening implementation (58.0%) or research (57.6%). Most agreed that ICSN helped to advance screening research and evaluation (75.4%), effective screening practices (71.2%), and screening policies (60.9%). Many reported that participating informed advances in their own research (68.7%) and screening implementation (50.2%) and policies (49.4%) in their settings. Approximately two-thirds agreed that ICSN helped advance career development among current experts (66.6%) and train the next generation (62.2%). Half (51.4%) reported that participating advanced their own careers. The 20 ICSN publications included 75 coauthors. They were cited in 589 publications with more than 2,000 coauthors.

**CONCLUSION:**

Findings provide evidence of the influence of ICSN on international knowledge dissemination, collaboration, and advances in cancer screening research, implementation, and policies and highlight the potential value of longstanding international research networks.

## INTRODUCTION

International research networks and consortia are accelerating advances in cancer science by facilitating international knowledge sharing, data harmonization and integration, and research collaboration. Through these approaches, these organizations are enhancing research rigor, enabling discoveries around rare events, and producing knowledge about the influence of multilevel interacting variables on health outcomes (eg, genetic, behavioral, community, and health system factors).^1-5^

Given these potential outcomes, new international networks and consortia continue to form, taking on new subjects and themes.^6,7^ However, these large research collaborations also require unique investments in the leadership, management, and conduct of the science—for example, leading development of a shared scientific vision and goals, facilitating communication, coordinating workflow, and establishing interoperability of data systems.^8^ These processes are critical to achieving scientific goals and also require additional investments of both financial and human resources.^9,10^

It is important to evaluate the outcomes and impacts of networks and consortia to better understand the added value of these approaches, in light of the resources required for their successful implementation. In 2018 to 2019, the National Cancer Institute (NCI) of the US National Institutes of Health evaluated the International Cancer Screening Network (ICSN). An NCI-supported international research network, the ICSN has been in continuous operation since 1988, making it one of the longest-standing international research networks. Evaluation findings are reported here, and implications are discussed for understanding the value of longstanding international research networks.

## METHODS

### The ICSN

The ICSN aims to reduce the global burden of cancer by advancing research that improves the reach and effectiveness of cancer screening.^11^ It pursues this aim by facilitating international knowledge sharing and research collaboration. The core activities of the ICSN are a biennial international scientific meeting and international scientific working groups. The ICSN is led and administered by the NCI. Biennial meetings and working groups are chaired by volunteer ICSN members.

The first ICSN meeting, held in 1988, was attended by an invited group of 24 cancer screening researchers from 11 countries who convened to discuss the potential for cross-national efforts to assess screening mammography diffusion and effectiveness.^11^ Since then, the ICSN has grown into a global cancer screening research network. The 2019 biennial scientific meeting was attended by 311 individuals from 37 countries.

ICSN biennial meetings are open to the cancer screening community and provide a forum for scientific discussion and dissemination of the most current cancer screening research methods and findings. Content emphasizes evaluating the effectiveness of screening for cancer sites where screening has been documented to reduce mortality (breast, colorectal, cervical, and lung).

ICSN scientific working groups identify key scientific priority areas in cancer screening and conduct international collaborative research on these topics. Currently active working groups are conducting research on international mammography screening skills, failures in the cervical cancer screening process, longitudinal adherence to fecal immunochemical testing for colorectal cancer screening, and overdiagnosis. Former ICSN working groups have conducted research on screening participation rates, health communication, performance evaluation, quality assurance, and other key topics. For a list of working group publications, see Appendix Table A1.

### Study Design

In 2018, the US NCI evaluated the ICSN to assess the outcomes and impacts of this long-running international research network. Research questions were:

- What are ICSN participants’ professional experiences and interests in the cancer screening field? and
- What have been the outcomes and impacts of the ICSN for:
  ○ Participants’ knowledge of cancer screening research and implementation;
  ○ Knowledge sharing, networking, and collaboration in the cancer screening field;
  ○ Participants’ professional activities in cancer screening;
  ○ Training and career development for the cancer screening community;
  ○ Cancer screening research and evaluation; and
  ○ Cancer screening implementation and policy?

The study design included a survey of ICSN participants and a bibliometric analysis of ICSN-produced publications. The survey was fielded in the spring of 2018 to 665 individuals who had participated in the 2015 and/or 2017 ICSN biennial meetings and/or who had subscribed to the ICSN listserv. The survey invitation was sent via e-mail and included an embedded link to the online self-administered survey. To incentivize participation, five survey respondents were randomly selected to receive a US$100 gift card.

The survey instrument included 43 questions (Data Supplement) in two sections. Section 1 addressed respondents’ professional experiences and interests in cancer screening and their attendance at ICSN meetings. Section 2 addressed the remaining research questions. The survey instrument was developed on the basis of formative interviews conducted by one of the coauthors (A.L.V.) with 14 current and former members of the ICSN Steering Committee. In addition, members of the ICSN Steering Committee and the ICSN directors at the NCI (coauthors S.H.T. and D.M.P.P.) reviewed drafts of the instrument. It was then pretested with global health professionals and finalized. Quantitative responses were analyzed in SAS (SAS Institute, Cary, NC) and qualitative responses were analyzed in Excel (Microsoft, Redmond, WA). The National Institutes of Health Office of Human Subjects Research Protections approved this study.

The bibliometric analysis addressed the impact of ICSN on international research collaboration and dissemination of knowledge in cancer screening. Bibliometric analyses examined coauthor country affiliations and coauthorship networks of ICSN-produced publications and citing publications. The base set of ICSN-produced publications was composed of articles attributed to the ICSN or an ICSN working group in the coauthor list or the acknowledgments section. Web of Science was used to identify citing articles through 2018. Income levels for coauthors’ countries were classified on the basis of the Atlas method defined by the World Bank.^12^ The Science of Science Tool was used to analyze coauthor data, and Gephi was used to draw coauthorship networks.

## RESULTS

### ICSN Participants’ Professional Experiences and Interests

A total of 265 individuals completed the first part of the survey (39.8% response rate), and 243 individuals completed the full survey (36.5% response rate). Just under half had 10 or fewer years of experience in the cancer screening field (46.5%), and the remaining respondents had 11 or more years of experience (Table 1). Most respondents expressed interest in breast (74.3%), bowel/colorectal (61.9%), and/or cervical cancers (57.4%). They worked primarily at academic institutions (37.4%) and government institutions (33.6%). Approximately two-thirds (64.5%) spent more than 25% of their work time conducting cancer screening research, and approximately a third spent more than 25% of their work time on policy development or advocacy (35.1%) or quality assurance (31.3%), respectively.

**TABLE 1:**
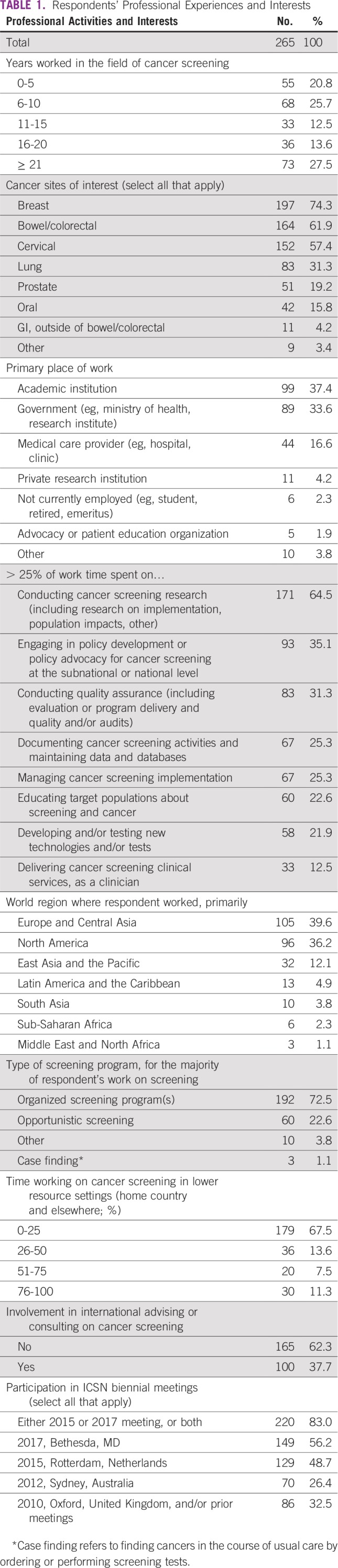
Respondents’ Professional Experiences and Interests

When asked what country they worked in, primarily, respondents named 95 countries in all seven world regions.^12^ Just more than one third worked primarily in Europe and Central Asia (39.6%) or North America (36.2%), respectively, and the remaining quarter (24.2%) were from the other five world regions combined. Approximately one third (32.5%) spent more than 25% of their work time focused on cancer screening in lower-resource settings in their home country or elsewhere. Approximately three quarters (72.5%) did most of their cancer screening work in the context of organized screening programs.

All respondents had participated in one or more ICSN biennial meetings. Most (83.0%) attended the 2015 and/or 2017 meeting. Approximately one quarter (26.4%) participated in the 2012 meeting, and approximately one third (32.5%) participated in prior meetings, where attendance was capped (2010) or by invitation only (2008 and earlier).

### Impacts of ICSN Participation on Knowledge Acquisition

Respondents were asked whether participating in the ICSN had helped to advance their knowledge of a range of professional activities in cancer screening (Table 2). Three quarters (75.7%) agreed that participating had helped to advance their knowledge regarding conducting cancer screening research, and approximately half agreed that participating had helped to advance their knowledge regarding conducting quality assurance (56.8%), engaging in policy development or advocacy (56%), managing cancer screening implementation (47.7%), and developing and/or testing new technologies/tests for cancer screening (47.7%). Only approximately a fifth (22.2%) agreed that participating had helped to advance their knowledge regarding delivering cancer screening services, as a clinician.

**TABLE 2:**
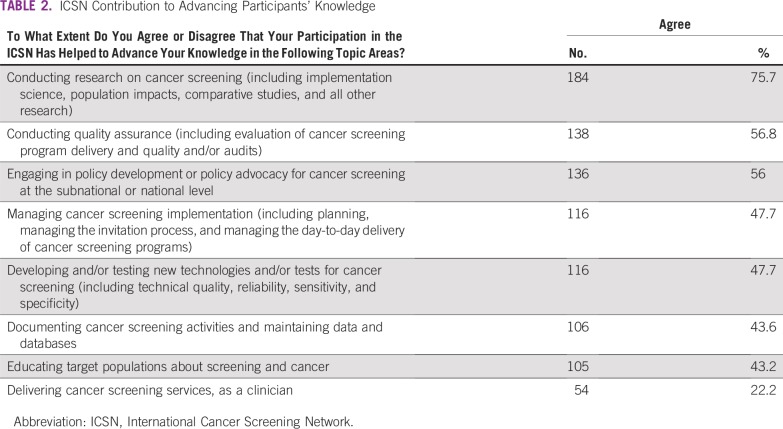
ICSN Contribution to Advancing Participants’ Knowledge

Responses to an open-ended question about whether participating in the ICSN had advanced their knowledge in other topic areas highlighted four key topics: cancer risk, including risk prediction modeling; risks of screening, including overdiagnosis and adverse events; principles of and key issues in screening for specific cancer sites, including those within and outside of one’s area(s) of expertise; and knowledge of the range of screening practices internationally.

### Impact of ICSN on International Knowledge Sharing, Networking, and Collaboration

Most respondents agreed that ICSN facilitated knowledge sharing and networking among diverse participants, including individuals engaged in cancer screening research and implementation (79.9%), living and working on different continents (74.0%), focused on different cancer sites (73.7%), and working in both high- and low-resource settings (62.2%). In addition, approximately three quarters (74.9%) of respondents agreed that ICSN has helped to form new international collaborations among cancer screening researchers, and approximately half (56.4%) agreed that ICSN had helped to form new international collaborations among cancer screening implementers (Table 3).

**TABLE 3:**
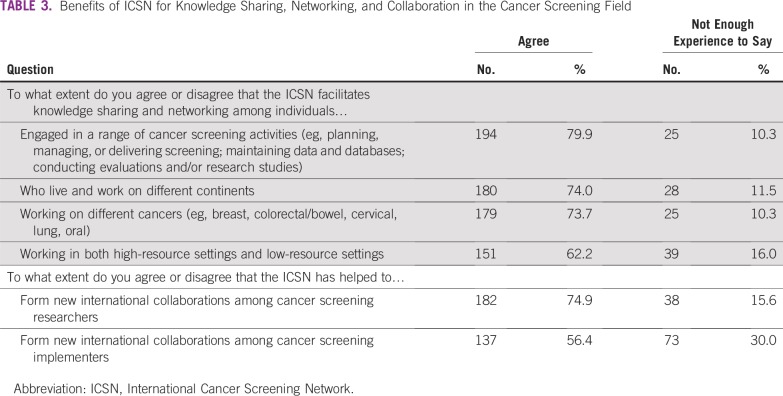
Benefits of ICSN for Knowledge Sharing, Networking, and Collaboration in the Cancer Screening Field

Most respondents also reported that participating in the ICSN produced benefits for them, personally, regarding knowledge sharing, networking, and collaboration (Table 4). They reported that participating had provided opportunities to contribute their knowledge and expertise to assist others (70.4%) and to form new collaborations related to cancer screening implementation (58.0%) and/or research (57.6%). Less than one fifth (18.1%) reported that participating had helped them to secure technical assistance for screening implementation.

**TABLE 4:**
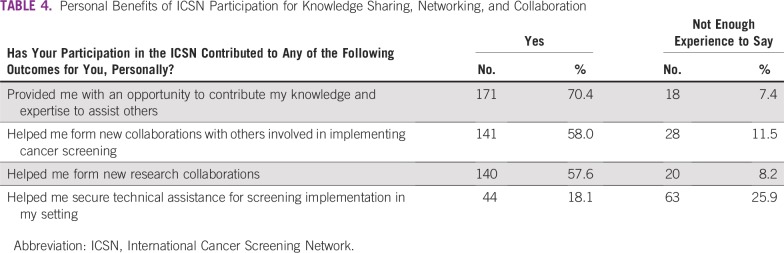
Personal Benefits of ICSN Participation for Knowledge Sharing, Networking, and Collaboration

### Impact of ICSN on Training and Career Development

Approximately two-thirds of respondents agreed that ICSN has helped to advance the career development of current cancer screening experts (66.6%) and train the next generation of cancer screening experts (62.2%). In addition, approximately half of respondents (51.4%) reported that participating in the ICSN had advanced their own career development.

### Impact of ICSN on Cancer Screening Research and Implementation

Approximately three quarters of respondents agreed that ICSN has helped to advance screening research and evaluation (75.4%), and nearly as many respondents (68.7%) reported that their own participation in the ICSN had informed advances in screening research or evaluation approaches and/or methods they used in their own work (Table 5).

**TABLE 5:**
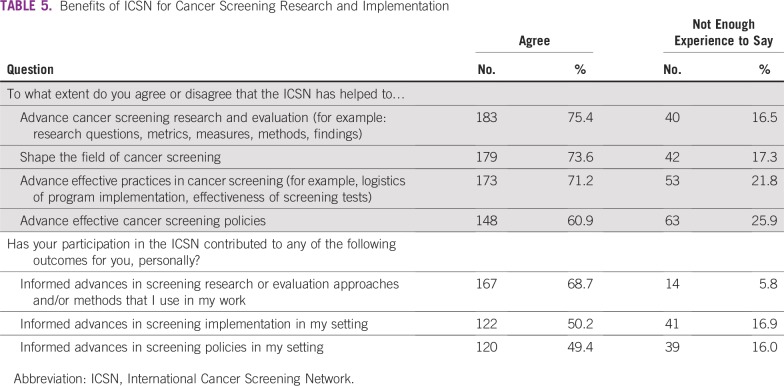
Benefits of ICSN for Cancer Screening Research and Implementation

Focusing on screening implementation, most respondents agreed that the ICSN had helped to advance effective practices in cancer screening (71.2%) and cancer screening policies (60.9%). In addition, approximately half of respondents reported that their participation in the ICSN had informed advances in screening implementation (50.2%) and policies (49.4%) in their own work settings. In an overall assessment of the value of the ICSN, nearly three quarters of respondents agreed that the ICSN had helped to shape the field of cancer screening (73.6%).

In open-ended comments, respondents attributed key successes of the ICSN—around facilitating knowledge acquisition, networking, collaboration, and advances in cancer screening research and implementation—in part to the design of the ICSN, including the network’s specialized focus on screening only, and the diversity of ICSN participants. In particular, they highlighted the value of including individuals from all over the world whose interests spanned cancer sites and whose professional activities, as a group, focused on both research and implementation. They also described how these characteristics enabled dissemination of effective policies and practices for cancer screening (Table 6).

**TABLE 6:**
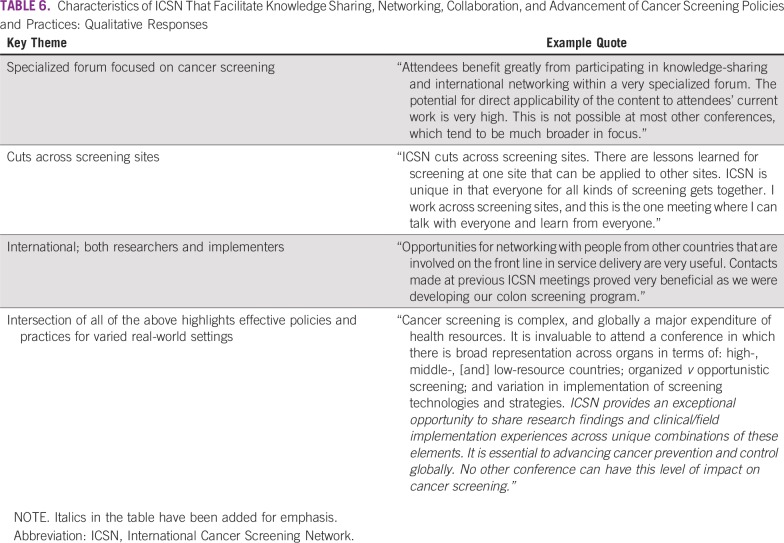
Characteristics of ICSN That Facilitate Knowledge Sharing, Networking, Collaboration, and Advancement of Cancer Screening Policies and Practices: Qualitative Responses

**FIG 1:**
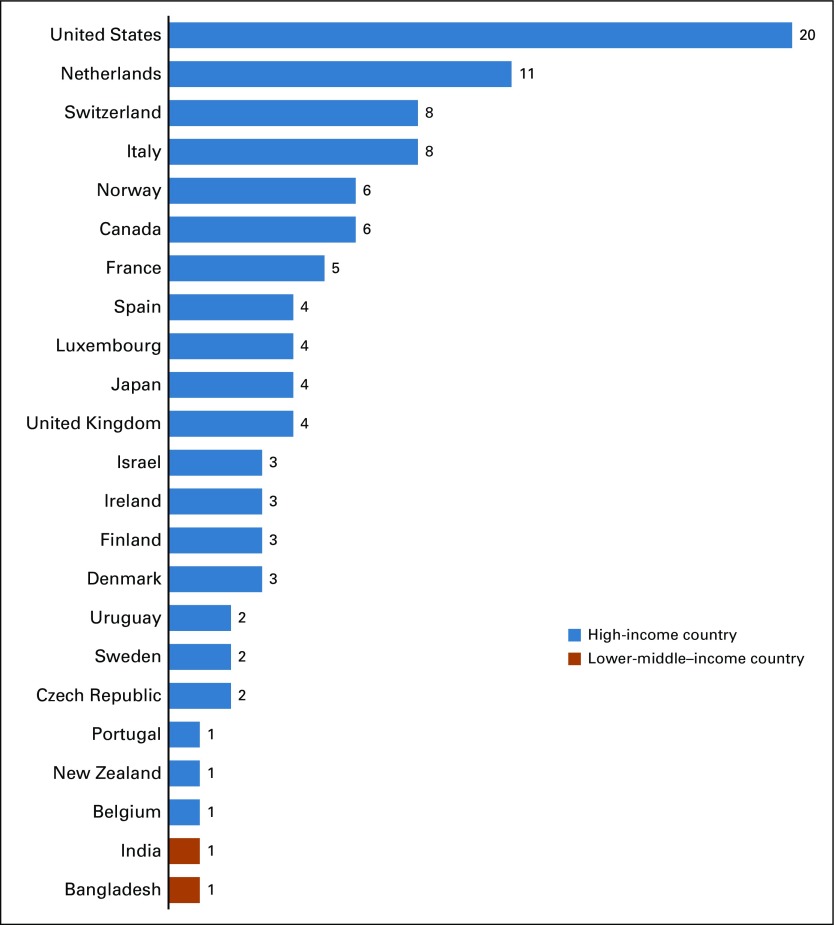
Countries represented among coauthors of International Cancer Screening Network–produced publications, by number of publications.

Bibliometric analyses identified 20 publications produced by ICSN scientific working groups and the ICSN leadership group (Appendix Table A1). These publications included a total of 75 coauthors located in 23 countries. This included 21 high-income countries (HICs) as well as two lower-middle income countries, both of which were included in only one of the 20 publications (Fig 1).

**FIG 2:**
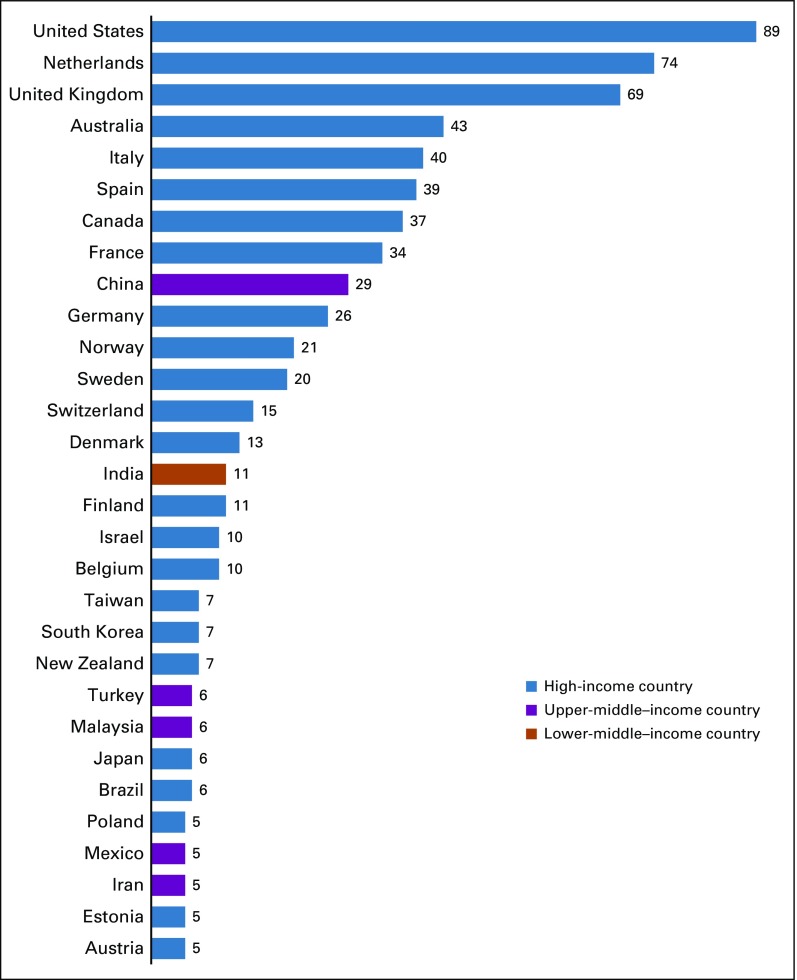
Countries represented among coauthors of citing publications, by number of citing publications. A cutoff point of five or more citing articles was used for this figure. As such, the figure does not represent all 589 citing publications and all 57 countries represented among coauthors of these citing publications.

These 20 publications were cited in 589 other publications, which together had more than 2,000 coauthors located in 57 countries. These included 39 HICs and 18 low- and middle-income countries, of which 14 were upper-middle–income countries and four were lower-middle–income countries. Of these 589 citing publications, 16 were other ICSN publications. Figure 2 shows the 30 countries that were represented in five or more citing publications. The remaining 27 countries were represented in fewer than five publications each.

**FIG 3:**
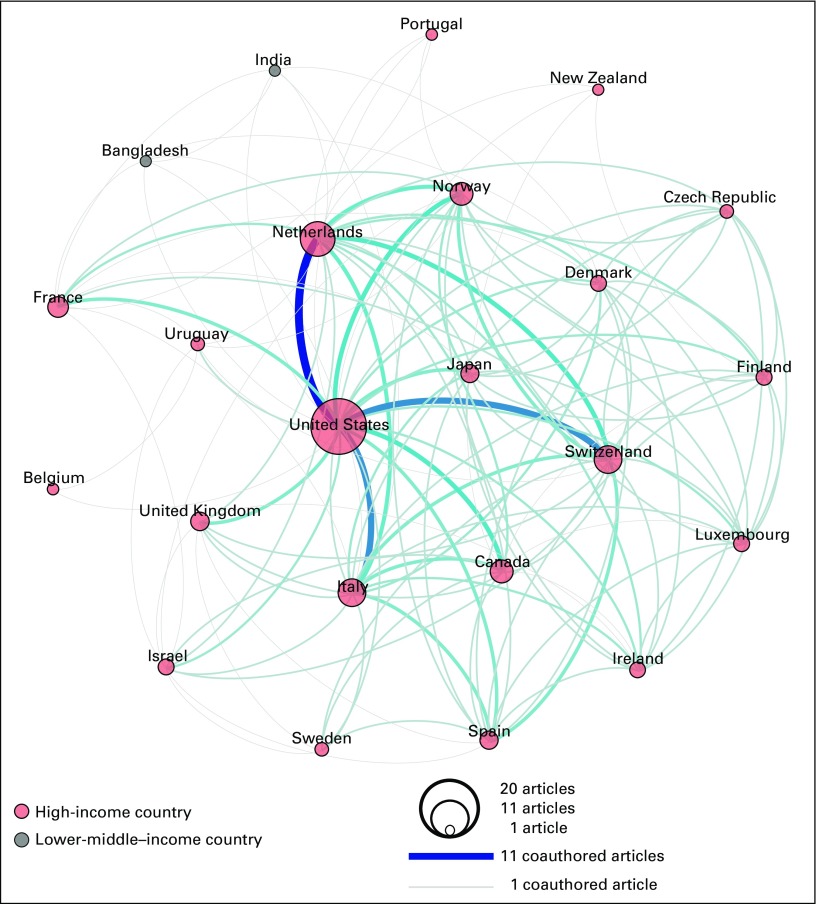
Each circle in the network graphic represents a country represented among the coauthors of the 20 International Cancer Screening Network publications. The size of a circle is proportional to the number of publications in which the country is represented. The color of a circle represents the country’s income level (World Bank Atlas method of classification). The lines connecting the circles represent coauthorships, with a gray line representing one coauthorship and a dark blue line representing 11 coauthorships, which was the maximum number found. The color gradient between gray and dark blue represents the range from one to 11 coauthorships.

The coauthorship network diagram for ICSN publications depicts collaboration patterns among countries represented by coauthors of the 20 ICSN publications, in conjunction with country income categories (Fig 3). It highlights the relative frequency of coauthorships among represented countries, ranging from one to 11 coauthored articles. The countries in the top half of this distribution are: the United States, the Netherlands, Switzerland, Italy, and Canada (Fig 3).

**FIG 4:**
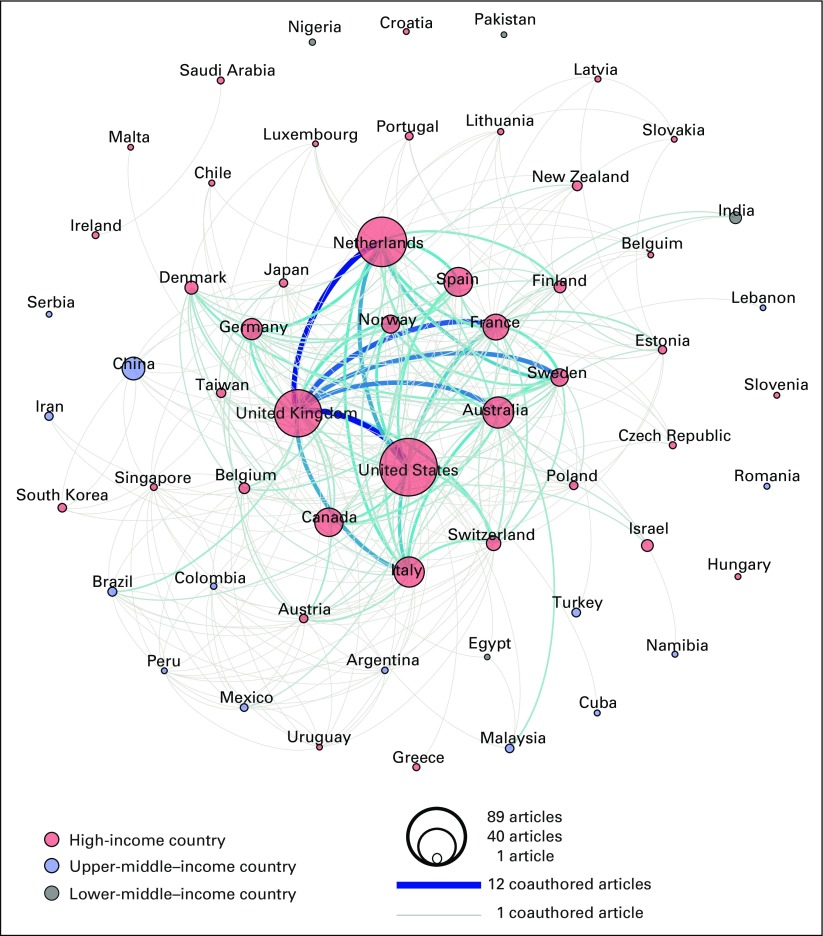
Each circle in the network graphic represents a country represented among the coauthors of the 589 citing publications. The size of a circle is proportional to the number of publications in which that country is represented. The color of a circle represent the country’s income level (World Bank Atlas method of classification). The lines connecting the circles represent coauthorships, with a gray line representing one coauthorship and a dark blue line representing 12 coauthorships, which was the maximum number found. The color gradient between gray and dark blue represents the range from one to 12 coauthorships.

The coauthorship network diagram for citing publications likewise highlights collaboration patterns, this time among countries represented among citing publication coauthors (Fig 4). It shows a range of one to 12 coauthored articles. The countries in the top half of this distribution include, once again, the United States, the Netherlands, Italy, and Canada, as well as the United Kingdom, Norway, Australia, Spain, France, Germany, China, and Sweden. In the citing articles’ network map, collaborations across country income levels are now visible.

## DISCUSSION

The current evaluation of the ICSN provided an opportunity to assess a 30-year-old international research group for participants, the science, and translational applications. In doing so, it addressed the question: what is the value of longstanding investments in international research collaboration?

Findings reflect ICSN participants’ strong professional focus on cancer screening research, as expected given the research focus of the network (Table 1). They also document that participants vary in their research, evaluation, and implementation activities, cancer sites of interest, and country locations—enriching the knowledge exchange that occurs via the ICSN (Table 1).

Survey results documented the contributions of the ICSN to knowledge acquisition, with particularly strong agreement that ICSN advanced participants’ knowledge of conducting cancer screening research, conducting quality assurance, and engaging in policy development or advocacy (Table 2). Topics on which respondents were less likely to agree that ICSN advanced their knowledge were those that focused on screening implementation, technology development, education of target populations, and delivery of clinical services. This pattern generally aligns with reported professional time spent on these activities and reflects the research focus of the network (Table 1).

There was strong agreement that ICSN facilitated knowledge sharing and networking across diverse participants and helped to form new international collaborations among screening researchers and implementers (Table 3). This was corroborated by reported personal benefits of ICSN participation (Table 4). Although few respondents reported that ICSN participation helped them secure technical assistance, nearly three quarters reported that it helped them contribute their knowledge and expertise to assist others (Table 4). This may reflect the fact that most participants in the survey were located in HICs (Table 1).

There was strong agreement that the ICSN has helped to advance cancer screening research and evaluation, shape the field of cancer screening, advance effective screening implementation, and advance effective screening policies (Table 5). Reported personal benefits of participation, and benefits of participation to one’s work setting, reinforced these findings (Table 5). The network diagram for citing publications also highlights the influence of the ICSN on the field of cancer screening, as evidenced by citations of ICSN publications in nearly 600 publications with coauthors from 57 countries—many more than the 23 countries represented by coauthors of ICSN publications (Figs 3 and 4).

Bibliometrics showed that the ICSN working groups and leadership group produced 20 publications. This modest number reflects the fact that publications are only one component of ICSN’s activities. In addition, many of these publications report on complex multinational comparative research requiring years to implement. The citing publication data show that ICSN publications, although few in number, have had an important influence on the field in both HICs and low- and middle-income countries (Fig 4). Furthermore, ICSN may have benefits beyond those addressed in this evaluation. For example, participants may have developed research questions, study designs, and/or publications that were inspired by topics, methods, or questions they learned about via ICSN.

Qualitative findings attributed the effectiveness of the ICSN to the combination of its specific focus on cancer screening and the diversity of participants’ professional experiences and knowledge within this field—across cancer screening sites, countries, high- and low-resource settings, and both research and implementation (Table 6). Participants emphasized that this research exchange helped to create evidence-informed practice and policy.

A key limitation of this evaluation was that the sampling frame excluded individuals who have not attended ICSN, who may have different perceptions of the network’s value. In addition, bibliometrics documented, to some extent, the impact of the ICSN on the screening literature but cannot measure impact on cancer screening implementation. Finally, this evaluation lacked a comparison group.

Future evaluations of other research networks and consortia might answer questions such as: How do these groups influence translational outcomes, and what outcomes do they influence? How do voluntary networks, funded networks, and funded portfolios of investigator-driven research compare in their influence on the science and translational applications? And how do varied approaches to the leadership, management, and administration of these groups, as well as varied scientific goals (eg, basic, translational), influence outcomes?

Recent scientific trends are amplifying the potential of international research consortia and networks to advance screening research. We are therefore likely to see an increase in these approaches. Evaluation of these initiatives is essential to fully understand their value. Findings from this evaluation suggest the potential for longstanding international research networks to contribute to knowledge sharing and collaboration and advancement of research methods and findings, related policies and practices, and fields of science.

## Figures and Tables

**TABLE A1:**
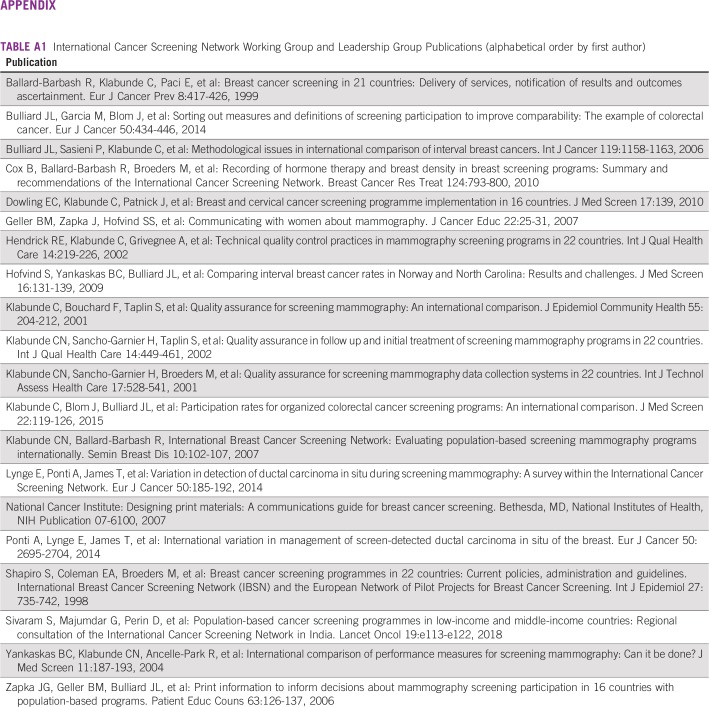
International Cancer Screening Network Working Group and Leadership Group Publications (alphabetical order by first author)
